# Feasibility and costs of water fluoridation in remote Australian Aboriginal communities

**DOI:** 10.1186/1471-2458-7-100

**Published:** 2007-06-08

**Authors:** Jonathon P Ehsani, Ross Bailie

**Affiliations:** 1 Formerly Victorian Public Health Training Scheme, 50 Lonsdale Street, Melbourne, Victoria, Australia; 2 Menzies School of Health Research and Institute of Advanced Studies, Charles Darwin University, Tiwi, NT 0810, Australia

## Abstract

**Background:**

Fluoridation of public water supplies remains the key potential strategy for prevention of dental caries. The water supplies of many remote Indigenous communities do not contain adequate levels of natural fluoride. The small and dispersed nature of communities presents challenges for the provision of fluoridation infrastructure and until recently smaller settlements were considered unfavourable for cost-effective water fluoridation. Technological advances in water treatment and fluoridation are resulting in new and more cost-effective water fluoridation options and recent cost analyses support water fluoridation for communities of less than 1,000 people.

**Methods:**

Small scale fluoridation plants were installed in two remote Northern Territory communities in early 2004. Fluoride levels in community water supplies were expected to be monitored by local staff and by a remote electronic system. Site visits were undertaken by project investigators at commissioning and approximately two years later. Interviews were conducted with key informants and documentation pertaining to costs of the plants and operational reports were reviewed.

**Results:**

The fluoridation plants were operational for about 80% of the trial period. A number of technical features that interfered with plant operation were identified and addressed though redesign. Management systems and the attitudes and capacity of operational staff also impacted on the effective functioning of the plants. Capital costs for the wider implementation of these plants in remote communities is estimated at about $US94,000 with recurrent annual costs of $US11,800 per unit.

**Conclusion:**

Operational issues during the trial indicate the need for effective management systems, including policy and funding responsibility. Reliable manufacturers and suppliers of equipment should be identified and contractual agreements should provide for ongoing technical assistance. Water fluoridation units should be considered as a potential priority component of health related infrastructure in at least the larger remote Indigenous communities which have inadequate levels of natural fluoride and high levels of dental caries.

## Background

Up until the 1980s, Indigenous Australian children were recognised as having better oral health than their non-Indigenous counterparts [1-4]. However recent evidence suggests that Indigenous children now have on average twice as much (and in some communities, up to five times as much) tooth decay as their non-Indigenous counterparts [5-7].

The efficacy of fluoride in the prevention of dental caries is incontrovertible [8-11]. Water fluoridation has been confirmed as the most cost-effective and socially equitable way of preventing dental decay in children and adults, providing 20–40% reductions in dental caries [12,13] and a number of recent international reviews [14-16] support the effectiveness of water fluoridation. Socio-economically disadvantaged groups, such as those living in remote Australian Indigenous communities stand to benefit the most from public health measures such as water fluoridation [17-21].

Although Australia has a high percentage of the population covered by water fluoridation one-third of Australians still do not have access to the benefit of this measure. The majority of Australians without access to fluoridated water live in rural Australia and are more likely to be from households of lower educational level and income [18]. A number of major recent national reports have included proposals to extend water fluoridation in rural areas of Australia [22-24], and Australia's National Oral Health Plan recommends roll out of fluoridation of water supplies to all Indigenous communities of over 1000 people[25].

Although population sizes as low as 1,000 people have traditionally been considered unfavourable for cost-effective water fluoridation [16], the cost of water fluoridation is reducing with the introduction of new technologies for water treatment and water fluoridation has been introduced for communities of less than 1,000 people [26]. Recent cost analyses have supported water fluoridation for communities of less than 1,000 people, especially vulnerable communities [27,28].

Many remote Indigenous communities could benefit substantially from the fluoridation of their water supplies. However, the small size and dispersed nature of these communities present significant challenges to the installation and maintenance of infrastructure, and there is a lack of empirical data on feasibility, cost and effectiveness. The aim of this paper is to address this gap through reporting on a study of the feasibility and costs of installation and operation of fluoridation units in two remote communities, X and Y, in the Northern Territory (NT) of Australia. Community X has a population of approximately 2,000 and the inhabitants of community Y number approximately 1,300. In community X, six year old dmft is 5.4 and 12 year old DMFT is 2.6. Community Y has a six year old dmft of 2.4 and 12 year old DMFT of 2.6.

## Methods

The water fluoridation trial involved the installation of two small scale fluoridation plants and prospective monitoring and evaluation over two years. The plants were designed to deliver fluoride to the water supply at 0.6 parts per million (mg/L) and are designed to shut down automatically in instances where fluoride levels exceed 1.0 mg/L [29]. Fluoride probes in the plant continuously sample the main water supply to provide monitoring and alarm functions and are designed to cause a shutdown in the event of overdosing.

The project involved the cooperation of NT government Department of Community Development, Sport and Cultural Affairs (DCDSCA), the Department of Health and Community Services (DHCS), the Power and Water Corporation (PWC), the Aboriginal and Torres Strait Islander Commission (ATSIC), the University of Adelaide and Menzies School of Health Research. Ethical approval for the project was provided by the Top End Health Research Ethics Committee.

The feasibility study used four data collection processes: monitoring of fluoride levels; semi-structured interviews with key informants; document review; and site visits.

### Monitoring of fluoride levels

Two parallel methods for data collection initiated from the inception of the trial were:

1. Daily recording of probe readings by community based ESO's

2. A system of remote monitoring of probe readings

In addition, ESOs were expected to undertake periodic sampling of water in the community distribution system to test the validity of the probe readings.

### Document review

Documentation pertaining to the maintenance and costs of the fluoridation plants was obtained with the permission of Power Water Corporation (PWC) Remote Operations senior managers. Cost figures for the operation of the plants were obtained from PWC records of invoices and accounts paid. Personnel involved in the fluoridation trial were required to keep a log of time dedicated to the trial in order to obtain an accurate estimate of management and labour costs. Additional cost information was provided by the equipment supplier. Data on plant functioning was also obtained from operational reports and communication records of implementing partners on the functioning of the plants. Costs are based on United States (US) dollars as of 2004/5.

### Semi-structured interviews

Interviews were conducted with key informants from the implementing partners of the trial including Power Water Corporation, Essential Services Officers (ESO's) and the NT Department of Primary Industry, Fisheries and Mines. Informants were selected on the basis of their experience and expertise with the implementation and operation of the plants. Interviews were recorded and transcribed, and sought respondents' perceptions of the challenges to the feasibility, sustainability and wider roll-out of water fluoridation plants in remote communities.

### Site visits

Site visits to the two water fluoridation plants were undertaken at commissioning and approximately two years later. During these visits the investigators observed plant functioning, and sought feedback from operational staff and community members on perceptions of the effective operation of the plants.

A draft of a full feasibility report was provided to the equipment suppliers, PWC and NT Government stakeholders for comment and their feedback has been incorporated into the final report and into this paper.

## Results

### Plant performance

At the time of the feasibility study data collection the fluoridation plant in Community X had been operational for approximately 18 of the 25 months since commissioning. The plant in Community Y had been operational for 22 of the 28 months since commissioning.

ESOs were required to report daily fluoride values from Monday to Friday of each week. For the purpose of estimating fluoride levels for the trial period, values for weekends were estimated by taking the mean of the previous and following week. Where data for ten days or less was not provided, the values for the preceding week of readings and the subsequent week of readings were averaged. Periods of greater than ten days where fluoride levels were not provided were left incomplete. The number of days on which the fluoride values were estimated using the above method for Community X was 126 (35%) and 4 for Community Y (1%).

Fluoridation plants in Community Y and Community X operated for a period of 758 and 708 days respectively. However, faulty probe readings plagued both sites for the first 12 months of the project. This problem was rectified with the installation of new probes from an alternative supplier in December 2004 in Community Y and January 2005 in Community X. As a result, data from probe readings for the first twelve months could not be used to accurately estimate the level of water fluoridation. The probes were considered to be accurately recording fluoride levels for 393 days (52% of the trial) in Community Y and 247 days (35% of the trial) in Community X. During this period fluoride levels were between 0.2 and 0.73 mg/L in Community Y and between 0.2 and 0.89 mg/L in Community X. Remote monitoring data from PWC were also intended to be used but no data from this system were available at the completion of the trial.

Fluoride levels for Community Y are estimated to have been within the recommended range (0.6 mg/L +/- 0.1) for at least 3% of the total period of the trial. Fluoride levels in Community X are estimated to have been within the recommended range for at least 28% of the total period of the trial. Incomplete recording of fluoride levels mean make it impossible to determine if water was fluoridated within guideline levels during the remainder of the trial period when plants were operating. Details of plant operation and fluoride dosing performance are presented in Table 1.

**Table 1:** Performance Indicators for Fluoridation plants in Community Y and Community X

**Overall Plant Performance**	**Community X**	**Community Y**
Number of days in trial	708	758
Number of days of operation with functioning probes (as a percentage of total number of days)*	247 (35%)	393 (52%)
Number of days of operation with malfunctioning probes (as a percentage of total number of days)*	318 (45%)	236 (31%)
Number of days plant not operational (as a percentage of total number of days)	143 (20%)	129 (17%)
Number of days fluoride estimated to be within recommended range (0.6 mg/L +/-0.1)*	198 (28%)	19 (3%)
Number of days fluoride < 0.6 mg/L *	54 (8%)	19 (3%)
Number of days fluoride > 1.0 mg/L	0	1 (identified by field testing)
Number. of plant shutdowns	3	3
Number of times fluoride > 1.0 mg/L caused a plant shutdown	0 (Community X was shut down as a precautionary measure following a high reading in Community Y)	1
Reasons for plant shutdowns	■ Faulty probe readings detected by field testing ■ Probe replacement ■ Fluoride accumulating on the base of the tank	■ Faulty probe readings detected by field testing. ■ Probe replacement
Reasons for fluoride levels falling outside specified range	■ Faulty probe readings	■ Faulty probe readings

* Values and percentages based on reported values and interpolations where data were missing for 10 days or less.

### Operational issues

#### Faulty fluoride monitoring probes

The original probes in the fluoridation plants were found to be under-reporting the levels of fluoride in the water supply during routine validation testing. Replacement probes were provided by the original supplier and were found to have the same fault. PWC contracted another supplier (ProMinent) to provide replacement probes. The original supplier subsequently decided to equip all new small scale fluoridation plants with Prominent probes acknowledging the standard probes were faulty.

#### Fluoride bags partially undissolved

To overcome the health risks associated with inhalation of fluoride dust the sodium fluoride is packaged in sealed, soluble food grade bags of 5 kg capacity. When placed in the water in the preparation tank the bags are intended to totally dissolve within 2–5 minutes [29]. In practice, the bags did not dissolve completely, leaving fragments of plastic in the saturation tank, blocking the circulation pumps, accumulating on the base of the tank and inhibiting the injection of fluoride into the water supply. Manual removal of the plastic from the tank presented health and safety challenges to the ESOs.

To address this problem the ESO in Community X manually opened each bag and added the contents into the water creating health and safety concerns. He complained of headaches and nausea after delivering fluoride to the tank using this method. These symptoms have been documented as early signs of acute fluoride toxicity [30,31], and were also experienced by a member of the evaluation team observing the practice of manual addition of fluoride powder to the tank during a site visit.

The failure of the bags to dissolve is considered to have been related to the age of the PVA bags (with some of the bag stock supplied possibly being over 2 years old) and extended periods of high storage temperature. The supplier has since reduced inventory to overcome the age issue and recommend that storage environments be considered with regard to temperature. The shipping containers also do not provide an ideal environment for storage.

Early water quality data indicated that the water should not be scale forming in the presence of fluoride. However, during the trial a layer of fluoride was found to be accumulating and compacting at the base of the saturation tank in Community X.

#### Fluoride scaling

The fluoride plants in Community Y and Community X used a down flow principle. That is, the saturated fluoride solution flowed down through the base of the tank and passed into a collection column from which the metering pumps draw. This down flow caused compaction or 'scaling' of the undissolved fluoride at the base of the tank. This was found to inhibit the flow of saturated fluoride solution to the dosing injection pumps. The plumbing of the saturation tank in Community X was altered to increase the recirculation in the saturation tank to promote dissolution of the fluoride. This problem remained unaddressed in Community Y at the time of our investigation. The supplier has since redesigned the plants to operate on an up flow system, thus eliminating the compaction.

#### Air in fluoride injection pumps

The compaction of fluoride at the base of the tank inhibited solution flow. Once the solution level fell below a certain level, air would enter the system and cause the pumps to stop operating. The ESO in Community X would manually 'bleed off' the air in the pumps and restart the system of fluoride injection. This issue has been addressed by the redesign of new fluoridation plants to operate on an upward flow principle.

#### Air conditioning in the fluoridation plants

Each fluoridation plant came preassembled in a shipping container. The container was fitted with a small air conditioner. The purpose of the unit is to keep the instrumentation cool and provide comfort for those working in the units. In practice the containers heated up considerably and the air conditioning unit had little or no effect in maintaining a moderate temperature. As a result the containers became unsuitable environments to work in, especially for prolonged periods.

The supplier acknowledged that shipping containers were not ideal for the tropical environment; but were preferred for their portability (remote area use) and security which was considered of paramount importance. An outer protective sunshield to remove the direct heat load and increased air conditioning capacity may assist in reducing the temperatures in the containers. Purpose built facilities for the fluoridation equipment is an alternative to shipping containers for future plants.

#### Fluoride concentration monitoring

Each ESO was instructed to take daily readings of the fluoride level meter and to fax the weekly results to PWC in Darwin. Fluoride levels in Community X were collected for approximately 72% of the complete trial period, while levels at Community Y were reported for approximately 15% of the trial period.

In addition, PWC installed a remote fluoride level monitoring system from a separate supplier. This system is limited by its dependence on functioning telephone lines and electricity at the community level. In Community Y it was found that the fluoride monitoring probes were not connected to the remote monitoring systems.

PWC staff did not regularly access the information and when requested to provide information on the functioning of the fluoridation plants for the period of the trial they found the computer system had not retained any information. The loss of the data on the operation of the plants from the remote communication equipment has limited the quality of data available for the trial. The remote monitoring equipment was infrequently checked and the data were lost at the completion of the trial. The supplier commissioned with responsibility to provide a backup of the data reported to have deleted the data from their servers by the time of the completion of the trial.

### Management issues

#### Powerwater corporation

During the course of the fluoridation trial, operational management responsibility for the fluoridation plants at PWC changed three times. The lack of continuity led to a loss of corporate knowledge in relation to the operation of the plants. A training session was provided in Darwin on the functioning and operation of the fluoride plants prior to shipment to the sites, in order to provide a base level of training for the local operations staff and all other interested parties. However, training for subsequent managers was limited or non existent and general knowledge of the operational side of the plants was not effectively shared. PWC operational personnel expressed frustration with the complexity of the plant operation and the lack of adequate training, *'I've received no training on this issue and when something goes wrong it is very difficult to know what is really going on'*.

#### Essential services officers

A lack of sufficient orientation to the purpose and operation of the plants was also identified as a problem by ESOs: *'The unit was put here and we were just supposed to monitor it, but if it's not working, it's a long way away to get someone here. If things were going wrong we'd make the decision to turn it off.' *This statement also identifies the lack of adequate role descriptions and delineation of responsibilities in relation to the plants.

ESOs were required to report daily fluoride values from Monday to Friday of each week and were not required to check readings on weekends or public holidays. These reports were not consistently provided. Managers at PWC stated the ESO in Community Y had minimal interest in the plant and subsequently did not undertake routine duties. The ESO did not take ownership of the plant and saw the trial as an additional burden to his work. In contrast, the ESO at Community X accepted responsibility for operating the plant, regularly reporting fluoride levels and alerting PWC to potential problems. The ESO in Community Y reported fluoride readings for 77 of the 758 days of operation (15% of the period required to be reported) and the ESO in Community X has reported readings for 346 of the 708 days of operation (72% of the period required to be reported).

### Economic costs

As seen in Table 3, total actual costs for the two year trial of the plants in Community Y and Community X were US$150,935 and US$154,036 respectively. The difference in operational costs between the communities is due to the additional maintenance work carried out on the plant in Community X and the labour charged by the ESO.

**Table 3:** Cost breakdown of plant installation and maintenance (USD)

**Total trial costs**	**Community Y**	**Community X**
■ Installation and start up costs	128,700	128,700
■ Operational and maintenance costs over first two years of operation	22,235	25,336

■ **Total**	**150,935**	**154,036**

Given the specific challenges associated with the trial subsequent plants may be less costly. The current cost for fluoridation plants housed in shipping containers similar to those in Community Y and Community X is estimated to be US$71,200 (inclusive of GST). The cost of a system shipped to Darwin for installation in an existing or purpose built facility was quoted at US$57,846 (inclusive of GST). Installation and commissioning cost at Community Y and Community X is estimated at US$12,760 (inclusive of GST). However, installation and commissioning costs may vary subject to site specific requirements.

Future implementation cost estimates presented in Table 4 are provided for two options: 1) housing the plant in a shipping container; or 2) housing the plant in a purpose built structure (the recommended option). DPIFM estimated the cost of a purpose built cyclone coded tin shed on a concrete slab with insulation, air venting and electric fan to be US$23,500.

**Table 4:** Estimated annual costs for future options (USD)

**Annual Estimates**		
Option	Capital	Recurrent

1 × Operational plant in shipping container	83,961	11,717
1 × Operational plant housed in purpose built structure	94,042	11,717

## Discussion

The trial of fluoridation of water supplies in two remote communities in the Top End has identified technical, operational and policy issues that need to be addressed for successful implementation of fluoridation of remote community water supplies.

Operational issues identified by the trial include the need to establish clear management lines and clear monitoring and reporting processes to ensure the effective and safe operation of the plants. Ongoing training on the operation and safety hazards of the water fluoridation plants should be conducted for all levels of operational staff. Implementing partners and operational staff involved in the fluoridation of public water supplies would benefit from orientation to the scientific basis and the population benefit of water fluoridation. Position descriptions, work plans and management process should also be updated to address effective operation of the fluoridation plants. In addition, reliable manufacturers and suppliers of equipment should be identified and contractual agreements should provide for ongoing technical assistance.

The deficiencies in the data on fluoride levels appear to result from inadequate personnel and data management and lack of familiarity with the electronic data collection system. Provision of ongoing training and support in the system of remote monitoring for PWC staff responsible for fluoridation plants and ensuring greater accountability from contracted suppliers to provide the necessary outputs should ensure improved data management processes. The role of the ESOs to monitor fluoride levels, address problems and alert PWC when issues arise was also found to be integral to the effective operation of the plants.

The capital costs for each of the two fluoridation plants in the trial was estimated to be about US$130,000, with operational and maintenance costs of about US$11,800 per year. These costs may change with refinements in technology and operation of plants, with economies of scale associated with wider implementation and with the need for permanent structures to house the plants. In any event, the actual costs are likely to be lower than for the trial and small in relation to the overall budget for remote community infrastructure in the study context. The marked difference in costs between the existing plants and future plants may be explained by the equipment upgrades and installation costs required by the trial. The design modifications to the plants by the supplier are expected to eliminate the additional costs incurred for the trial plants. Nevertheless, budgeting for future plants should account for site specific requirements and contract variations.

In response to the submission of this study to the Northern Territory Government the Minister for Health clarified the policy and funding responsibilities within and between governments for this type of public health intervention, including systems of management and accountability. The Cabinet of the Northern Territory Government determines the wider implementation of fluoridated water supplies for remote communities. The DHCS is responsible for the development of the fluoridation policy and the Department of Planning and Infrastructure and PWC are responsible for the funding and ongoing operational management of the plants. The Minister also committed to the implementation of the structural modifications recommended in the study and the establishment of an enhanced management process. At the completion of a further 12 month review and subject to a satisfactory outcome, the wider implementation of fluoridated water supplies will be considered.

## Conclusion

Evidence of effectiveness of water fluoridation in preventing dental caries from other settings, and local cost effectiveness analyses indicate fluoridation of at least the larger remote communities will be a good investment in terms of health benefits. Australian governments should consider the implementation of water fluoridation units as a priority component of health related infrastructure in remote Indigenous communities which have inadequate levels of natural fluoride and high levels of dental caries. With a relatively small investment, the fluoridation of public water supplies of remote communities and towns should result in significant improvements in dental health of community residents in the medium to long term, with important flow on effects to general health.

## Competing interests

The author(s) declare that they have no competing interests.

## Authors' contributions

JE conducted most of the fieldwork and data analysis for this study and took primary responsibility for drafting the manuscript. RB was responsible for the conceptualisation, establishment and general management of the study, contributed to the interpretation of the findings and to preparation of the manuscript. Both authors read and approved the final manuscript.

**Table 2:** Summary of issues and responses

**Issue**	**Impact**	**Response**
Fluoride probes	Under reporting of fluoride levels	More reliable probes now standard in new plants
Fluoride bags partially undissolved	Fluoride saturation and injection inhibited	Mesh baskets to collect undissolved bags now standard in new plants
Fluoride scaling	Fluoride saturation and injection inhibited	Redesign of flow circulation to prevent scaling – now standard in new plants
Air in injection pumps	Pumps cease operation	Redesign of flow circulation to prevent air injection – now standard in new plants
Air conditioning in containers	Environment uncomfortable and unsuitable temperature for instruments	Future plants to be installed in more permanent structures which provide better insulation from heat
Fluoride concentration monitoring	Ineffective system	New supplier commissioned to install monitoring equipment

## Pre-publication history

The pre-publication history for this paper can be accessed here:


